# Virus and Autoantigen-Specific CD4+ T Cells Are Key Effectors in a SCID Mouse Model of EBV-Associated Post-Transplant Lymphoproliferative Disorders

**DOI:** 10.1371/journal.ppat.1004068

**Published:** 2014-05-22

**Authors:** Stefanie Linnerbauer, Uta Behrends, Dinesh Adhikary, Klaus Witter, Georg W. Bornkamm, Josef Mautner

**Affiliations:** 1 Clinical Cooperation Group Pediatric Tumor Immunology, Children's Hospital, Technische Universität München, Munich, Germany; 2 Helmholtz Zentrum München, Munich, Germany; 3 German Centre for Infection Research (DZIF), Munich, Germany; 4 Laboratory of Immunogenetics, Ludwig-Maximilians-Universität, Munich, Germany; Baylor College of Medicine, United States of America

## Abstract

Polyclonal Epstein-Barr virus (EBV)-infected B cell line (lymphoblastoid cell lines; LCL)-stimulated T-cell preparations have been successfully used to treat EBV-positive post-transplant lymphoproliferative disorders (PTLD) in transplant recipients, but function and specificity of the CD4+ component are still poorly defined. Here, we assessed the tumor-protective potential of different CD4+ T-cell specificities in a PTLD-SCID mouse model. Injection of different virus-specific CD4+ T-cell clones showed that single specificities were capable of prolonging mouse survival and that the degree of tumor protection directly correlated with recognition of target cells *in vitro*. Surprisingly, some CD4+ T-cell clones promoted tumor development, suggesting that besides antigen recognition, still elusive functional differences exist among virus-specific T cells. Of several EBV-specific CD4+ T-cell clones tested, those directed against virion antigens proved most tumor-protective. However, enriching these specificities in LCL-stimulated preparations conferred no additional survival benefit. Instead, CD4+ T cells specific for unknown, probably self-antigens were identified as principal antitumoral effectors in LCL-stimulated T-cell lines. These results indicate that virion and still unidentified cellular antigens are crucial targets of the CD4+ T-cell response in this preclinical PTLD-model and that enriching the corresponding T-cell specificities in therapeutic preparations may enhance their clinical efficacy. Moreover, the expression in several EBV-negative B-cell lymphoma cell lines implies that these putative autoantigen(s) might also qualify as targets for T-cell-based immunotherapy of virus-negative B cell malignancies.

## Author Summary

The γ-herpesvirus Epstein-Barr virus (EBV) is associated with several human malignancies, including post-transplant lymphoproliferative disorders (PTLD) in immunocompromised patients. The successful treatment of EBV-positive PTLD by the infusion of EBV-specific T-cell lines has provided an important proof of principle for immunotherapy of EBV-associated tumors and for cancer immunotherapy in general. EBV-specific T-cell preparations for clinical application are generated by repeated stimulation with autologous LCL *in vitro.* These lines contain CD4+ and CD8+ components but the specificity of the infused CD4+ T cells is still poorly defined. Using a mouse model of PTLD, we assessed the antitumoral potential of single virus-specific CD4+ T-cell clones. While T cells specific for a virion antigen of the virus prolonged mouse survival, other virus-specific clones had no effect or, unexpectedly, even promoted tumor growth. Moreover, the principal antitumoral effectors in LCL-stimulated T-cell preparations were CD4+ T cells specific for non-virus antigens. The definition of virion- and potentially autoantigen-specific CD4+ T cells as key effectors against PTLD may contribute to the design of generic and standardized protocols for the generation of T-cell lines with improved clinical efficacy. In addition, the observed tumor-promoting propensity of some CD4+ T cells may have implications for adoptive T-cell therapy in general.

## Introduction

About 20% of all human cancers are caused by pathogens and of these 80% by viruses [1]. The viral proteins expressed in these tumors represent neo-antigens and potential targets for immunotherapeutic approaches [2]. The oncogenic Epstein-Barr virus (EBV), a member of the gamma-herpes virus family, has been implicated in the pathogenesis of several human malignancies of lymphoid and epithelial origin [3]. Acquired orally, EBV persists lifelong in the human host by establishing latency in B cells but is normally contained as an asymptomatic infection by T-cell surveillance. Consequently, patients with T-cell immunodeficiency are at heightened risk of developing EBV-associated malignancies [3]. In immunosuppressed hematopoietic stem cell transplant (HSCT) recipients, such EBV-positive post-transplant lymphoproliferative disorders have been successfully treated by the infusion of polyclonal EBV-specific T-cell preparations that are generated by repeated stimulation of peripheral blood T cells with autologous EBV-infected B cells (LCL) *in vitro* and contain CD8+ and CD4+ T-cell components [4]–[6].

Despite its proven safety and remarkable efficacy, adoptive T-cell therapy still has a limited role in the management of virus-associated complications in transplant recipients, mainly because of the logistical and financial implications that are associated with extensive *in vitro* T-cell culture, as well as the time required to generate virus-specific T-cell lines when the clinical need is urgent. To expedite the preparation procedure, various protocols have been designed that aim at isolating effector populations directly from stem cell donors, including *ex vivo* selection of defined EBV antigen-specific T cells with pentamers [7], or cytokine secretion and capture technology [8], [9]. Moreover, the recently established repository of cryopreserved virus-specific T-cell lines from healthy seropositive donors provides partially HLA-matched, off-the-shelf products for adoptive transfer [10]. Given the difficulty of generating virus-specific T-cell lines from EBV-naive donors *in vitro*, recipients of stem cells from cord blood might particularly benefit from such allogeneic effectors [3], [5], [6]. Of note, the success of immunotherapy seen in HSCT recipients has not been matched in solid organ transplant (SOT) patients, most likely because the continuous immunosuppressive environment limits proliferation and persistence of adoptively transferred cells. Response rates in SOT recipients with refractory PTLD that were treated with autologous or allogeneic LCL-stimulated T-cell preparations were reported to range around 50% [5], [6]. Importantly, better clinical responses were observed when the infused T cells expressed a broad T-cell receptor repertoire [11], suggestive of a broadly targeted T-cell response, and when they contained higher proportions of CD4+ T cells [10], [11]. For unknown reasons, the CD4/CD8 T-cell ratio in LCL-stimulated T-cell lines can vary greatly [12], [13]. These findings imply that the clinical efficacy of T-cell preparations may be increased by tailoring its cellular composition and, in extension, antigen specificity. However, in contrast to the well-characterized EBV-specific cytotoxic CD8+ T-cell response [3], [14], relatively little is known about function and specificity of virus-specific CD4+ T cells. *Ex vivo* analyses of latent antigen-specific CD4+ T-cell memory has led to the identification of multiple epitopes, and virus carriers usually exhibit memory responses to several epitopes that are derived from more than one antigen [15]–[17]. For the few lytic cycle antigens examined to date, again multiple reactivities were detected per donor [18]–[20], indicating that the EBV-specific CD4+ T-cell response is broadly distributed across different latent and lytic cycle antigens. A similar pattern of antigen specificity was detected in LCL-stimulated T-cell preparations. Besides viral antigen-specific T cells, these lines also contain CD4+ T cells specific for cellular antigens, whose expression is probably up-regulated by EBV infection [20], [21]. The remarkable breadth of the virus-specific CD4+ T-cell response and the fact that classical PTLD, like LCL, express all latent antigens of EBV and contain lytically infected cells expressing ∼80 lytic cycle proteins [3], [22], raises the question, whether the different CD4+ T-cell specificities are equally tumor-protective or whether some have non-redundant functions in tumor control and, therefore, should be enriched in T-cell preparations for adoptive therapy.

Here, we used the well-established PTLD-SCID mouse model [23], [24], that permits to assess efficacy of T-cell preparations in a preclinical setting [25], to comparatively evaluate the tumor-protective potential of different CD4+ T-cell specificities *in vivo*.

## Results

### Induction of CD20+ EBV+ PTLD-like tumors in SCID mice by different cell types

**Figure 1:**
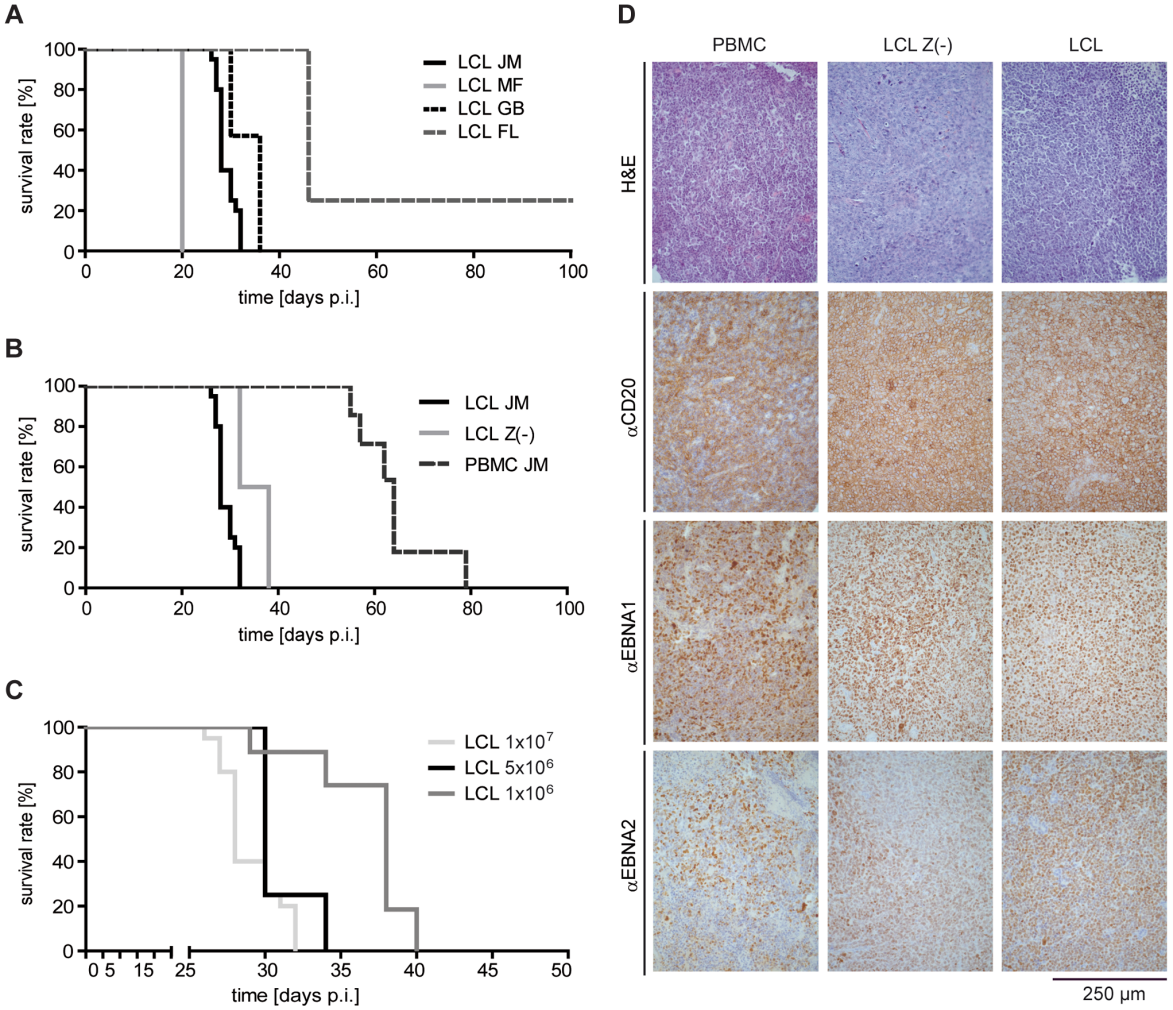
Induction of human PTLD-like tumors in immunodeficient mice. (A) Intraperitoneal injection of SCID mice with 1×10^7^ LCL of four different donors led to tumor development with an incidence of 75 – 100% and a latency of 20 and 46 days (group sizes: LCL FL and LCL MF n = 4; LCL GB n = 11; LCL JM n = 20; days p.i.: days post injection). (B) Injection of 1×10^7^ LCL, 1×10^7^ LCL Z(-), or 5×10^7^ PBMC from the same donor led to tumor development in all animals but with different latency (group sizes: LCL JM n = 20; PBMC JM n = 6; LCL Z(-) JM n = 6). All survival curves and donor dependent incidences and latencies were reproduced in several independent experiments. (C) Different numbers of LCL from the same donor were injected in mice and the survival determined. Results are depicted in a Kaplan-Meier curve (group sizes: 1×10^7^ n = 20; 5×10^6^ n = 4; 1×10^6^ n = 6; median survival 28, 30, and 38 days). (D) Developing tumors were confirmed as PTLD-like lymphomas. Formalin-fixed, paraffin-embedded tumor slides (3–8 µm) were stained with H&E (first row) and with antibodies against the human B cell marker CD20 (second row), as well as antibodies against the EBV latent proteins EBNA1 (third row) and EBNA2 (fourth row), whose co-expression is characteristic of PTLD.

To assess the tumor-protective potential of different T-cell populations in the PTLD-SCID mouse model [24], [26]–[28], mice were i.p. injected with 1×10^7^ LCL or 5×10^7^ PBMC from EBV-positive donors and tumor incidence, latency and localization analyzed. After injection of LCL, PTLD-like tumors developed with 100% incidence in three out of four cases (Figure 1A) with a latency of 20 to 46 days. Tumors usually developed with slightly delayed kinetics when LCL Z(-) of the same donor were injected (Figure 1B). Tumor latency was also extended when reduced numbers of LCL were injected (Figure 1C). Injection of PBMC from EBV-seropositive donors also led to tumor development but with much slower kinetics (Figure 1B). Tumors either formed below the liver and were then often connected with the porta hepatis, or were located at the injection site. Human origin and PTLD-like histology of the tumors was verified by measuring huIgG in mouse serum (data not shown) and by immunohistochemical analysis of tumor sections [29]. Although PBMC-induced tumors were more heterogeneous in their cellular composition, all tumors expressed human CD20 and the EBV-proteins EBNA1 and EBNA2 (Figure 1D).

### The CD4+ and CD8+ component of LCL-stimulated T-cell preparations have similar tumor-protective potential *in vivo*

**Figure 2:**
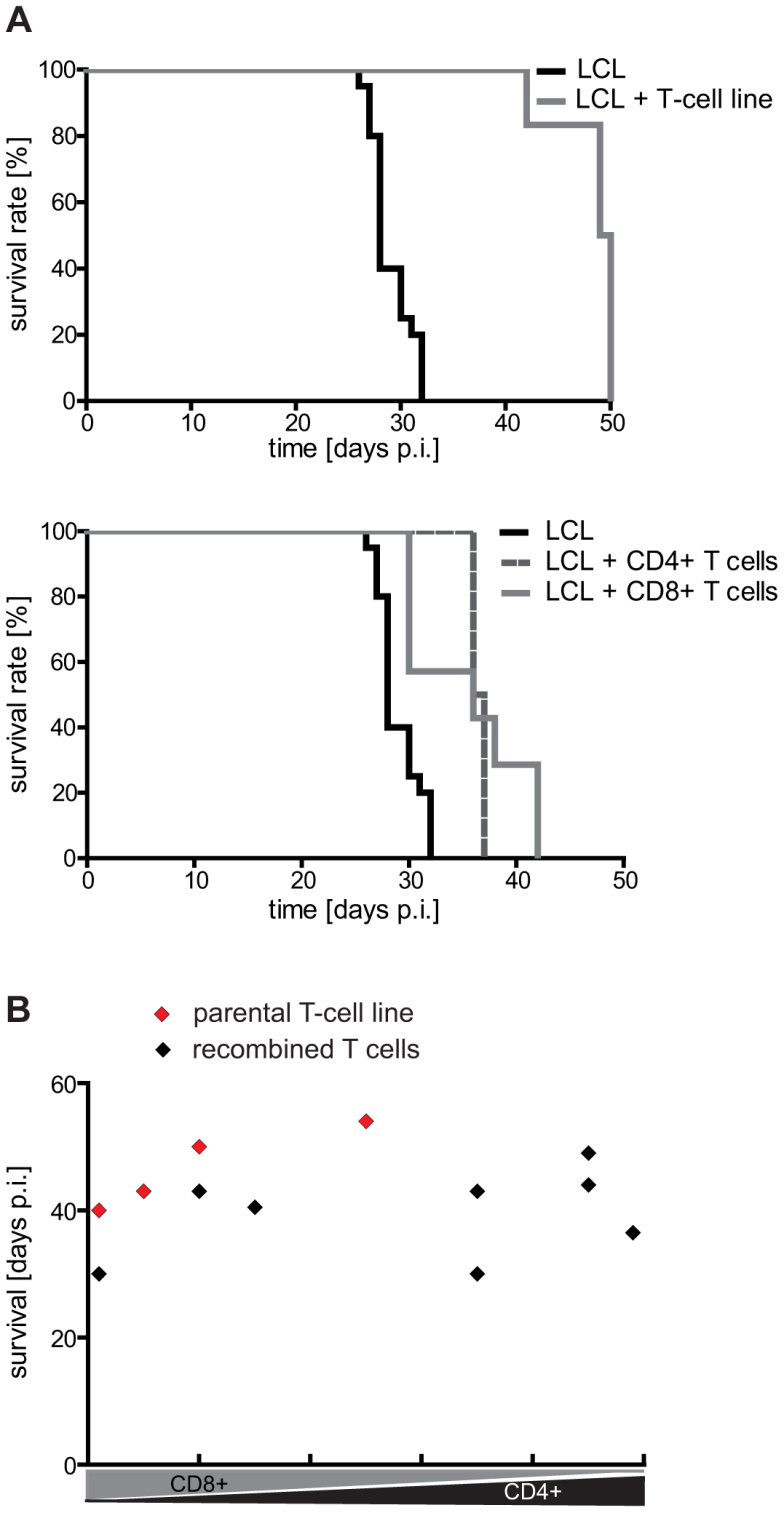
Analysis of the tumor-protective efficacy of CD4+ and CD8+ T cells *in vivo*. (A) Mouse survival after adoptive transfer of autologous LCL-stimulated T cells. Mice were i.p. injected with 1×10^7^ LCL followed by a separate injection of an equal number of the indicated T cells on the opposite side of the body. CD4+ and CD8+ T-cell populations prolonged mouse survival to a similar extent (LCL n = 20, LCL + T-cell line n = 6; LCL + CD4+ T-cell line n = 4; LCL + CD8+ T-cell line n = 7). (B) Tumor-protective potential of different CD4/CD8 T-cell combinations. Separated CD4+ and CD8+ T-cell populations were recombined at different ratios and tested as described in (A). No significant increase in efficacy was observed (group sizes: n = 4; p = 0.4457). Representative results from one out of three different donors are shown.

To compare the tumor-protective efficacy of CD4+ versus CD8+ T cells *in vivo*, T-cell lines were generated from several donors by four rounds of *in vitro* stimulation with autologous LCL and then separated into CD4+ and CD8+ subsets by MACS. Mice that had received 1×10^7^ LCL were i.p. injected on the same day with an equal number of the separated (n = 4–7), or, as control, the unseparated T cells (n = 6) on the opposite flank. Although T-cell preparations from different donors proved differently effective, mouse survival was consistently prolonged to the same extent by the CD4+ and CD8+ components (Figure 2A), indicating that both T-cell subsets possess similar tumor-protective capacity. Because the single components were not as efficacious as the parental T-cell line, and because T-cell preparations with higher CD4+ numbers had shown better clinical responses [10], CD4+ and CD8+ T-cell subsets were recombined at different ratios ranging from 0–100% CD4+ T cells, and tested in the same way. None of the combinations, including reconstituted CD4/CD8 ratios of the parental T-cell lines (group size n = 4), showed enhanced tumor protection (Figure 2B). These results suggested that the T-cell subsets have additive but not synergistic effects on mouse survival and that the comparatively lower tumor-protective effect of the subset combinations might have been due to an impaired fitness or vitality of the T cells following the separation procedure.

### Different EBV-specific CD4+ T-cell clones can have opposing effects on mouse survival *in vivo*

Given the remarkable breadth of the EBV-specific CD4+ T-cell response, we sought to investigate whether and to which extent single CD4+ T-cell clones were able to delay tumor growth, and whether tumor protection *in vivo* correlated with target cell recognition and inhibition of proliferation *in vitro* [19]. To this aim, different latent and lytic cycle antigen-specific CD4+ T-cell clones, that recognize and growth-inhibit unmanipulated LCL to various degrees *in vitro* [19], [30], were injected together with autologous LCL or PBMC and tumor latency analyzed.

**Figure 3:**
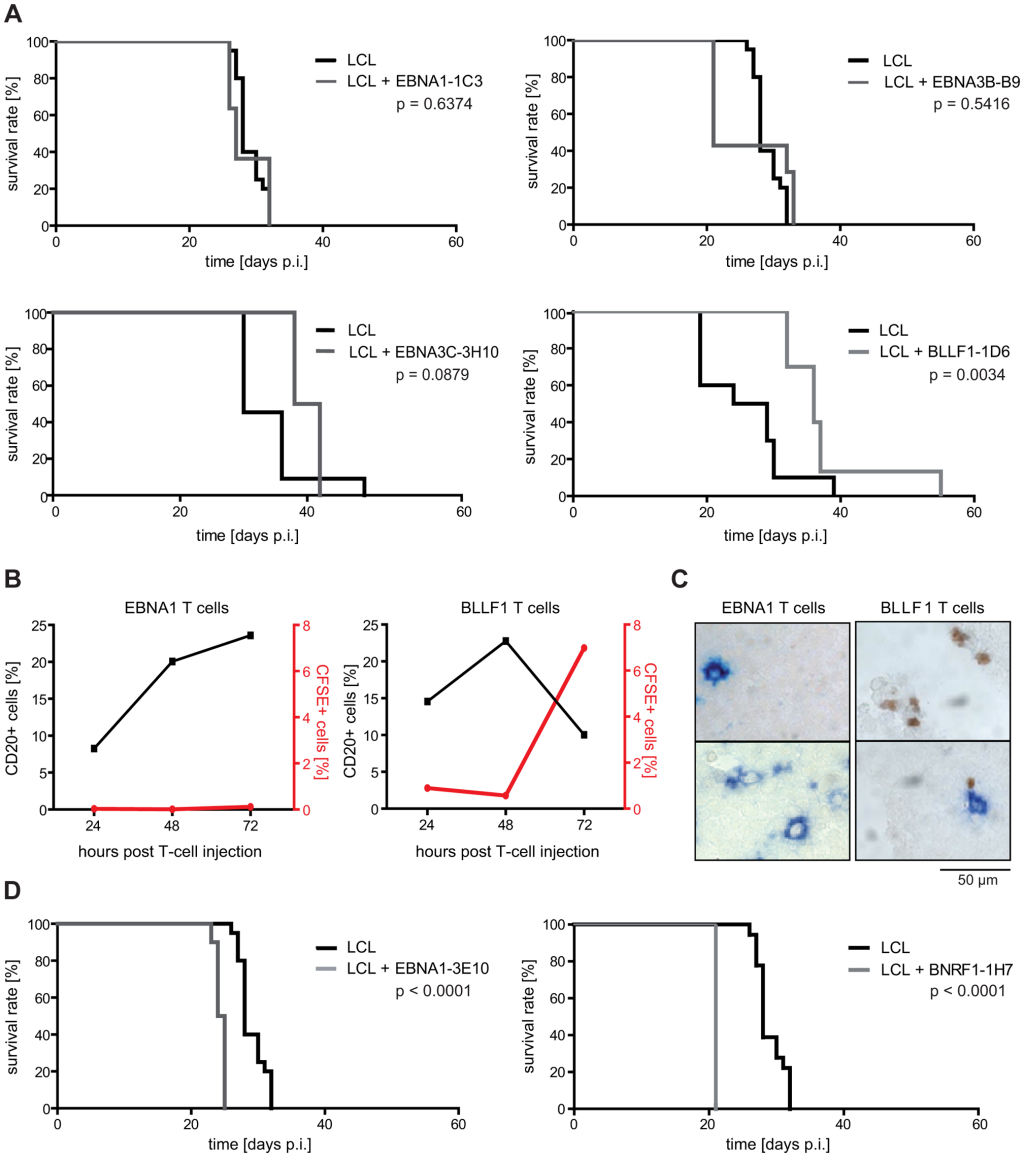
EBV-specific CD4+ T cells differ in their tumor-protective potential. (A) Survival of mice after adoptive transfer of different EBV-specific CD4+ T-cell clones. 1×10^7^ LCL and 1×10^7^ T cells were consecutively i.p. injected and mouse survival analyzed. As exemplified by the EBNA1-specific T-cell clone 1C3 and the EBNA3B-specific clone B9, injection of latent antigen-specific T cells had no effect on mouse survival, except for EBNA3C-specific CD4+ T cells that showed a trend towards delaying tumor growth (group sizes: EBNA1-1C3: LCL n = 20, LCL + T cells n = 11; EBNA3B-B9: LCL n = 20, LCL + T cells n = 7; EBNA3C-3H10: LCL n = 11, LCL + T cells n = 4). Adoptive transfer of the BLLF1-specific CD4+ T-cell clone 1D6 prolonged mouse survival (group sizes: LCL n = 10; LCL + T cells n = 10; summarized results of 2 independently performed experiments). (B) CFSE-labeled BLLF1- and EBNA1-specific T cells were i.p. injected into mice that had received autologous LCL 25 days before. Single cell suspensions of tumors were analyzed 24, 48, or 72 hours post injection by FACS for the presence of CFSE-labeled T cells as well as human CD20-expressing tumor cells. BLLF1- but not EBNA1-specific T cells infiltrated tumors and led to a decrease in the percentage of CD20+ cells. (C) Immunostaining of tumor sections from mice described in (B). Cryo-embedded tumor sections were double-stained with FITC- and BLLF1-specific antibodies to detect tumor infiltrated CFSE-labeled T cells (brown) and BLLF1-expressing tumor cells (blue). BLLF1-specific T cells infiltrated tumors and were found in proximity to antigen expressing cells while no EBNA1-specific T cells were found to infiltrate the tumors. Two immunostainings of two separate tumor sections are shown in each case. (D) Injection of the EBNA1-specific T-cell clone 3E10 and the BNRF1-specific T-cell clone 1H7 led to faster tumor development and shortened mouse survival (group sizes: EBNA1-3E10: LCL n = 20, LCL + T cells n = 10; BNRF1-1H7: LCL n = 20, LCL + T cells n = 4).

As shown for the EBNA1- and EBNA3B-specific T-cell clones 1C3 and B9 [30], T cells that failed to recognize unmanipulated LCL *in vitro* had no effect on mouse survival (Figure 3A). A possible correlation of *in vitro* and *in vivo* effector functions was also suggested by a slight, but statistically not yet significant prolongation of mouse survival by the EBNA3C-specific T-cell clone 3H10, which moderately recognized LCL *in vitro*. Consistent with these findings, tumor development was significantly delayed when the BLLF1-specific T-cell clone 1D6 was transferred, which recognized and growth-inhibited LCL very efficiently *in vitro* [19] (Figure 3A). BLLF1-1D6-treated mice showed a median survival benefit of 9.5 days, which is similar to mice that had received tenfold less LCL (1×10^6^) without T cells (Figure 1C). Moreover, similar results were obtained with this clone in the PBMC-SCID mouse model, but these experiments have not yet reached statistical significance due to the limited availability of large numbers of PBMC from individual donors (data not shown). These results indicated that single CD4+ T-cell specificities can significantly prolong mouse survival and that tumor-protection might correlate with target cell recognition and growth-inhibition *in vitro*. This notion was further supported by experiments in which 1×10^7^ CFSE-labeled BLLF1-specific or, as a control, EBNA1-specific cells were i.p. injected into animals that had received 1×10^7^ autologous LCL 25 days before and started forming tumors. Both groups of mice were sacrificed 24, 48, or 72 hours after T-cell injection and the tumors analyzed by FACS (Figure 3B) and immunohistochemistry (Figure 3C) for T-cell infiltration. BLLF1- but not EBNA1-specific T cells accumulated in tumors over time. Concomitantly, a reduction in the proportion of human CD20+ cells was observed (Figure 3B). Some BLLF1-specific T cells were even detected in direct contact with BLLF1-positive cells (Figure 3C), lending further support to a potential correlation of T-cell effector functions *in vitro* and *in vivo*.

However, infusion of the EBNA1-specific clone 3E10, that failed to recognize unmanipulated LCL *in vitro* [30], and the BNRF1-specific T-cell clone 1H7 (group size n = 10 and n = 4), that efficiently recognized and growth-inhibited LCL *in vitro* [20], accelerated tumor development (Figure 3D). When compared to the tumor-protective clone BLLF1-1D6, these T cells responded to their cognate antigen with similar affinity, secreted similar amounts and patterns of cytokines, and displayed similar cytolytic activity (Figure S1 in Text S1). These findings suggested that besides target cell recognition and lytic activity, still unknown functional differences among virus-specific CD4+ T cells may impact on antitumoral efficacy.

### Enriching virion antigen-specific CD4+ cells in T-cell preparations confers no additional survival benefit *in vivo*

**Figure 4:**
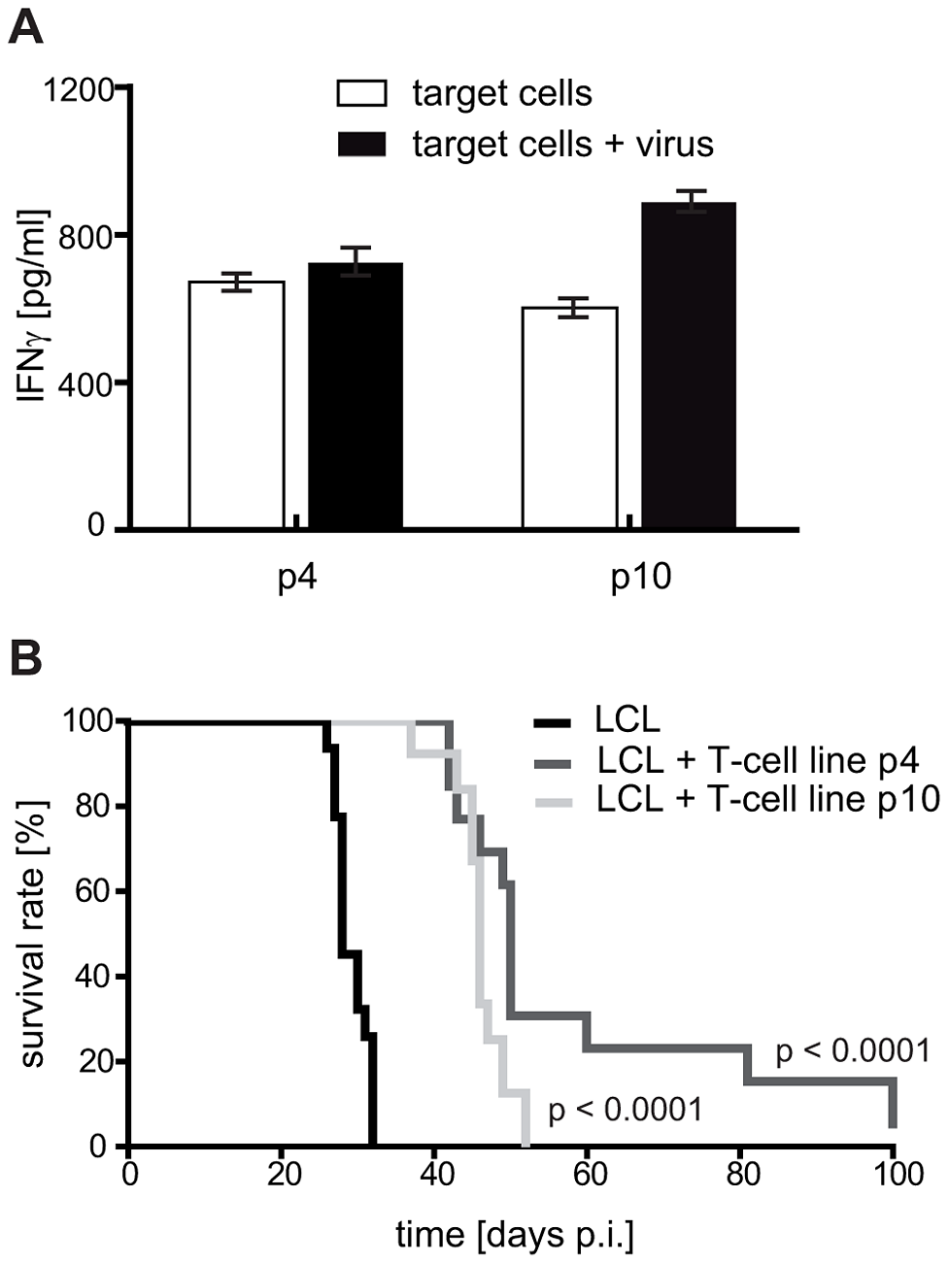
Later passage T-cell preparations show increased virion antigen specificity but are less tumor-protective. (A) Reactivity of the T-cell lines against virion antigens. With increasing numbers of stimulation, the T-cell lines progressively responded against virion antigens transferred by viral particles. T-cell specificity was tested by cytokine secretion upon stimulation with autologous LCL Z(-). The target cells were either left untreated or loaded with virus particles for presentation of structural antigens (mainly late lytic antigens). (B) Tumor protection by early and late passage T-cell lines. 1×10^7^ LCL and 1×10^7^ T cells stimulated with autologous LCL four (p4) or ten (p10) times *in vitro* were simultaneously injected into SCID mice (group sizes: LCL n = 20; LCL + T cells p4 n = 13; LCL + T cells p10 n = 13; depicted results are combined from two independently performed experiments). Later passage T-cell lines prolonged mouse survival less efficiently.

This tumor-promoting effect of some CD4+ T cells notwithstanding, the above described experiments suggested that tumor-protection *in vivo* correlates with T-cell recognition of target cells *in vitro*. Since virion antigen-specific CD4+ T cells efficiently recognize LCL *in vitro,* these results implicated CD4+ T cells specific for structural antigens of the virus as particularly tumor-protective. As demonstrated previously, the frequency of such T-cell specificities is usually low in early passage T-cell lines, but increases with further rounds of stimulation *in vitro* [20]. Accordingly, later passage LCL-stimulated T-cell lines might exhibit a higher tumor-protective potential. As expected, T cells from the same donor stimulated four or ten times *in vitro* both recognized autologous LCL *in vitro*, but responses against virus-pulsed LCL were more pronounced after ten rounds of stimulation. These results indicated that the proportion of T cells with virion antigen specificity had increased (Figure 4A and Figure S2 in Text S1). When tested *in vivo*, both T-cell lines prolonged median survival of LCL-injected animals to a similar extent; 50 days in the case of p4 (n = 13) and 46 days in the case of p10 (n = 13). Thus, despite an increased response against virus-pulsed target cells, later passage T-cell lines were not more efficacious *in vivo* (Figure 4B). In fact, the tumor-protective potential of these LCL-stimulated T-cell lines seemed to decline with the number of passages, either because extended *in vitro* culture impaired their antitumoral activity *in vivo,* as demonstrated for CD8+ T cells [31], and/or relevant specificities were lost.

### The tumor-protective effect of LCL-stimulated T-cell lines is mostly mediated by non-virion antigen-specific T cells

To investigate the antigen-specificity of protective T-cell lines in more detail, we generated T-cell lines by repeated stimulation with three different stimulator cells, (i) LCL cultured in media containing FCS (LCL-FCS), (ii) LCL cultured in media containing FCS and acyclovir (LCL-FCS-ACV), and (iii) LCL cultured in media containing human serum (LCL-HS) instead of FCS. ACV inhibits EBV late lytic gene expression and is used for safety reasons in clinical T-cell stimulation protocols to prevent virus production [32]. As verified in T-cell recognition assays, T-cell lines stimulated with LCL-FCS-ACV were devoid of virion antigen-specific T cells (Figure S3 in Text S1). LCL-HS were used as stimulators to investigate whether recognition of FCS-derived antigens presented on injected LCL by FCS-specific T cells contributed to tumor protection [13].

**Figure 5:**
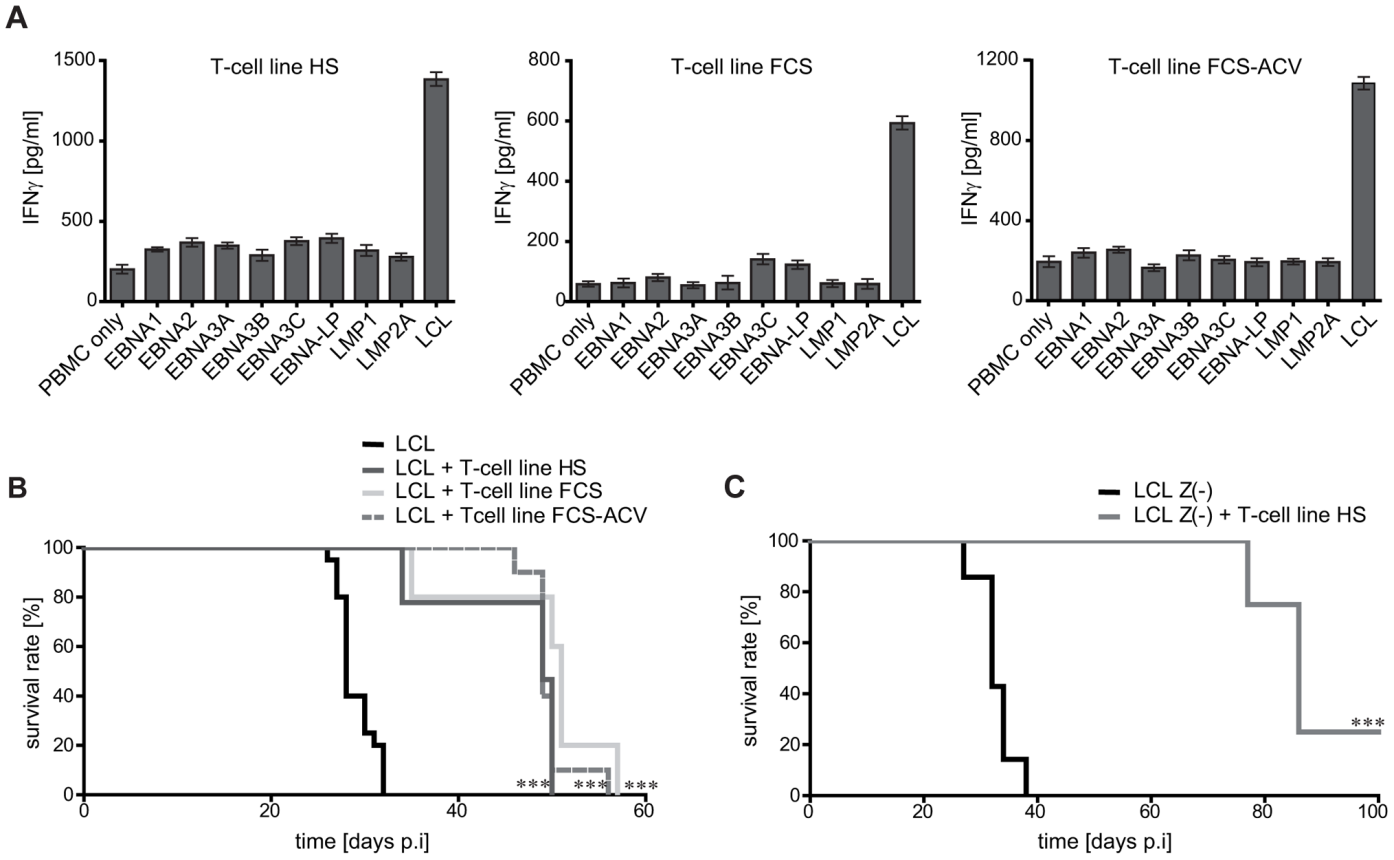
Mouse survival is mediated by non-virus-specific T cells. T-cell lines were generated by four rounds of stimulation *in vitro* with LCL cultivated in media containing human serum (HS), fetal calf serum (FCS), or fetal calf serum plus acyclovir (FCS-ACV) to prevent the expression and presentation of virion antigens. (A) Specificity analysis of the generated T-cell lines. Autologous PBMC were pulsed with recombinant EBV latent proteins [20] for 24 h and then probed with the T cells. Whereas all T-cell lines recognized LCL, none specifically responded against PBMC pulsed with any of the latent proteins of EBV. (B) Following i.p. injection with autologous LCL as tumor inducing cells, all three T-cell lines prolonged mouse survival significantly (*** p<0.0001). Group sizes: LCL n = 20; LCL + T-cell line HS n = 9; LCL + T-cell line FCS n = 12; LCL + T-cell line FCS-ACV n = 10 (summarized results of two independent experiments). (C) Injection of T-cell line HS together with LCL Z(-) significantly prolonged mouse survival, demonstrating that virion antigen-specific T cells are not required for the tumor protective effect. Group sizes: LCL Z(-) n = 7; LCL Z(-) + T-cell line HS n = 8 (*** p<0.0001).

Irrespective of the stimulator cells used, all three T-cell lines recognized autologous LCL *in vitro*, but failed to respond to autologous PBMC pulsed with recombinant EBV latent proteins (Figure 5A). Efficient processing and presentation of peptides derived from these recombinant proteins was confirmed using latent antigen-specific CD4+ T-cell clones (Figure S4A in Text S1). LCL-FCS and LCL-HS-stimulated T cells potentially recognized EBV lytic cycle antigens and/or autoantigens, whereas LCL-FCS-ACV-stimulated T cells might have been directed against cellular antigens and possibly immediate early and early lytic cycle antigens.

Surprisingly, *in vivo* all three T-cell lines significantly prolonged median mouse survival to approximately 50 days (group sizes n = 9-12) (Figure 5B). Thus, LCL-stimulated T-cell preparations that lacked virion antigen-specific T cells were not compromised in their antitumoral efficacy, indicating that tumor-protection was mediated by T cells specific for non-virion antigens.

To further substantiate this notion, 8 mice were co-injected with tumor-inducing cells that are unable to express lytic cycle antigens (LCL Z(-)) and T cells stimulated with LCL-HS as effectors. Although lytic cycle antigen-specific T cells, including virion antigen-specific T cells, were *a priori* ineffective in this experimental setting, mouse survival was significantly prolonged with three out of eight animals never developing any tumors (Figure 5C). No human IgG was detected in the serum of these mice (data not shown). Mice in this experiments survived on average for 86 days, compared to 32 days without T cells (n = 7) (Figure 5C). Although this remarkable protective efficacy might have been partly due to the slightly less aggressive nature of LCL Z(-) as compared to LCL-induced tumors (Figure 1B and [33]-[35]), these results clearly demonstrated a considerable therapeutic potential of LCL-stimulated T-cell lines independent of EBV latent or lytic cycle antigen recognition.

### CD4+ T cells specific for potential autoantigens prolong mouse survival

To more directly evaluate the antitumoral efficacy of non-viral antigen-specific T cells *in vivo*, T-cell lines were generated by stimulation with LCL Z(-) or miniLCL, thereby precluding the expansion of T cells that recognize EBV lytic cycle antigens. Following more than 30 rounds of stimulation, these T cells usually expressed one or few Vβ chains, suggesting that these lines were directed against one or few antigens (data not shown).

**Figure 6:**
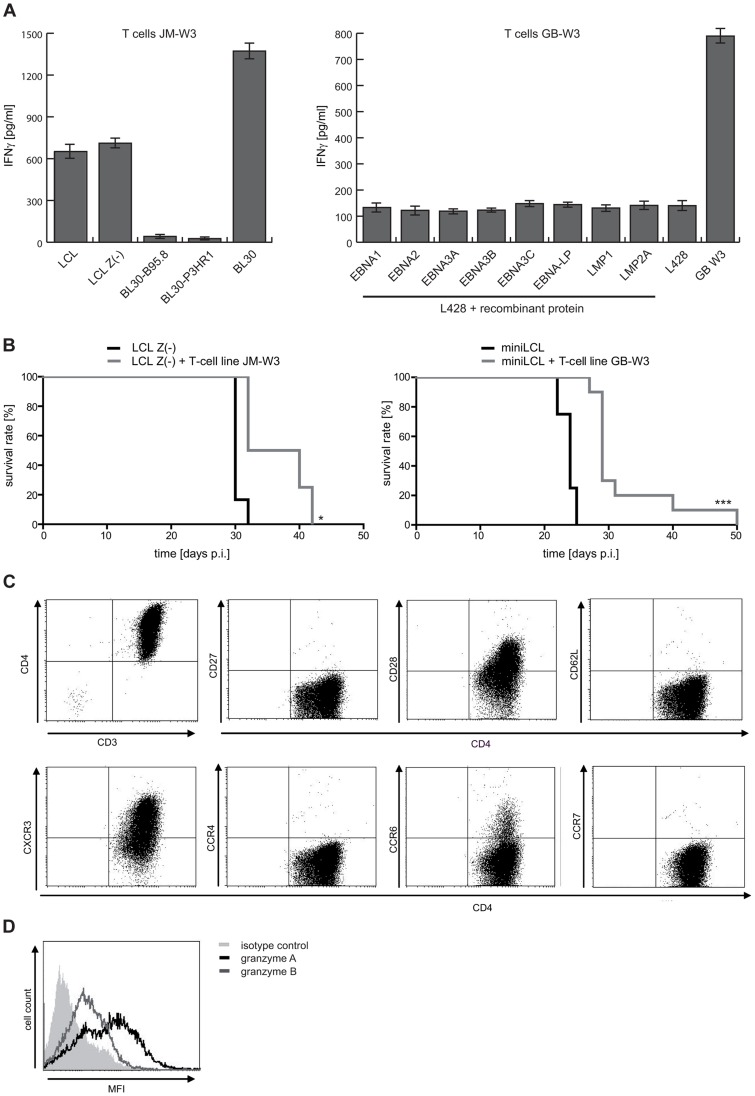
LCL Z(-)- as well as miniLCL-stimulated T-cell lines recognize autoantigens and prolong mouse survival. (A) Recognition of autoantigens by LCL Z(-) or miniLCL-stimulated T cells. Specificity analysis of the T-cell line JM-W3 was performed using autologous LCL and LCL Z(-) as well as HLA-matched EBV-negative and EBV-positive BL30 cell lines. T-cell recognition of the EBV-negative BL30 cell line, but barely of BL30 cells that had been infected with the B95.8 or the P3HR1 EBV strains, demonstrated that these T cells recognized a non-viral antigen(s). Recognition of viral antigens by the GB-W3 T cells was excluded by probing the cells with the HLA-matched, EBV-negative Hodgkin lymphoma cell line L428 pulsed with recombinant latent proteins of EBV. (B) Analysis of the tumor-protective potential of these autoreactive T-cell lines *in vivo*. 1×10^7^ LCL Z(-) or miniLCL were i.p. injected in combination with 1×10^7^ autologous T cells and tumor development assessed (LCL Z(-) n = 6; LCL Z(-) + T cells JM-W3 n = 4; miniLCL n = 4; miniLCL + T cells GB-W3 n = 10; * p<0.05; *** p<0.001) (C) Phenotypic characterization of the autoreactive T cells GB-W3 by FACS. Autoreactive T cells displayed a CD3+CD4+ effector T-cell phenotype (CD62L-CCR7-) of differentiated T cells (CD27-CD28+CXCR3+CCR4-CCR6+/-), and produced granzyme A (black line) and B (grey line) (D).

The miniLCL-stimulated T-cell line JM-W3 recognized autologous LCL and LCL Z(-), as well as the HLA-matched EBV-negative Burkitt's lymphoma cell line BL30. Recognition was not due to alloreactivity, because these T cells failed to recognize the EBV-positive convertants (BL30-B95.8 and BL30-P3HR1). Thus, this T-cell line recognized a differentially expressed cellular antigen(s), but not viral antigens (Figure 6A and Figure S4C-D in Text S1). In the case of the miniLCL-stimulated T-cell line GB-W3, reactivity against EBV latent antigens was excluded by assessing recognition of the HLA-matched, EBV-negative Hodgkin's lymphoma cell line L428 that had been pulsed with single recombinant EBV latent antigens (Figure 6A and Figure S4B in Text S1). Co-injection of 1×10^7^ JM-W3 or GB-W3 T cells together with 1×10^7^ autologous LCL Z(-) or miniLCL into SCID mice prolonged median mouse survival from 30 to 36 days in the case of JM-W3 T cells (group sizes LCL Z(-) n = 6; LCL Z(-) + T cells n = 4), and from 24 to 29 days in the case of GB-W3 (group sizes miniLCL n = 4; miniLCL + T cells n = 10), demonstrating that autoantigen-specific T cells were tumor-protective in this preclinical PTLD model (Figure 6B and Table S1 in Text S1).

Similar to virus-specific effectors, these putative autoreactive T cells displayed a differentiated effector/effector-memory Th1 phenotype [36], [37] (CD62L^−^, CCR7^−^, CD27^−^, CD28^+^, CXCR3^+^) (Figure 6C), that was confirmed by the expression of the cytotoxins granzyme A and B in these T cells (Figure 6D).

## Discussion

The identification of low endogenous CD4+ T-cell numbers as important risk factor for the development of EBV-associated diseases in immunosuppressed patients [38], and of better clinical responses in patients with PTLD receiving EBV-specific T-cell lines that contained higher proportions of CD4+ T cells [10], have implied an important role for CD4+ T cells in the control of EBV-driven lymphoproliferation. Thus, elucidating the role of CD4+ T cells in tumor defense may facilitate to generate T-cell preparations with enhanced clinical efficacy and to reduce the logistic complexity of this form of immunotherapy that still precludes its application outside specialized academic centers [3]. The EBV-specific CD4+ T-cell response, albeit one to two orders lower in magnitude, appears to target a much broader set of viral antigens than the corresponding CD8+ T-cell response [6], [14], [20]. To investigate whether these numerous CD4+ T-cell specificities are functional redundant or fulfill complementary roles in tumor defense, we assessed their tumor-protective potential in a preclinical PTLD model.

In contrast to earlier [39], but in accordance with recently published data [40], CD4+ T cells in our LCL-stimulated preparations delayed tumor growth as effectively as the CD8+ components. Contrary to the above mentioned clinical experience, however, antitumoral efficacy was not affected by the CD4/CD8 ratio of the injected T-cell preparations. This functional redundancy implied that both components recognized PTLD-like tumors with similar efficiency. In patients, CD4+ T cells probably also exert indirect “helper” functions that remained undetected in this xenogenic model, where human T cells fail to persist long-term and complex immune networks are unlikely to form.

To assess functional differences among virus-specific CD4+ T cells we injected T-cell clones with defined specificities together with autologous LCL or PBMC from EBV-seropositive donors into SCID mice. Unexpectedly, the T-cell clones had divergent effects on mouse survival, ranging from tumor-protective in the case of BLLF1-specific T cells, to ineffective in the case of most latent antigen-specific T cells, to tumor growth-promoting in the case of EBNA1-3E10 and BNRF1-1H7. The correlation of tumor-protective but not tumor-promoting propensity of T cells *in vivo* with target cell recognition and inhibition of proliferation *in vitro* suggested that still unknown phenotypic differences may exist between these populations. Neither the pattern nor the amount of secreted cytokines, including paracrine growth factors like IL-6 that are known to shorten tumor latency in SCID mice [34], [35], [41], differed consistently among tumor-promoting and tumor-protective T cells (Figure S1 in Text S1, and data not shown). How certain CD4+ T cells promote tumor growth is still unknown, but given the potential clinical implications, warrants further investigation. This dichotomous function of CD4+ T cells may also provide an explanation for the contrasting effects of LCL-stimulated CD4+ T-cell lines on tumor growth in different studies [39], [40], and for the baffling observation that tumor development in SCID mice injected with primary B cells from EBV-positive donors depends on the presence of T cells [33].

Unexpectedly, EBNA1-specific CD4+ T cells had no or a tumor growth-promoting effect *in vivo*. This was surprising because EBNA1 peptide-selected T-cell preparations were successfully used in the clinic to treat PTLD [42]. The reasons for these discrepant results are currently not known. The clinically used T-cell preparations, however, contained CD4+ and CD8+ T-cell components and only about 60% of the adoptively transferred T cells were EBNA1-specific. Therefore, it cannot be excluded that tumor regression was mediated by EBNA1-specific CD8+ T cells and/or T cells with undefined specificities. An important role of CD8+ T cells in the control of PTLD has been implicated by clinical studies using peptide or MHC class I pentamer-selected T-cell preparations [7], [8]. The infused T cells were predominantly CD8+ and were directed against different viral antigens. Collectively, these studies point towards a redundant function of different latent or lytic antigen-specific T cells in the control of PTLD in stem cell transplant recipients. However, in solid organ transplant recipients, response rates are generally lower (around 50%) and positively correlate with the CD4+ T-cell content of the infused T-cell preparations [10], suggesting that in these patients, CD4+ and CD8+ T cells do not have completely redundant antitumoral functions. Whether virus-specific CD4+ T cells, including those directed against EBNA1 as well as other viral antigens, that had no effect on tumor growth in the SCID mouse model, are of therapeutic importance in this cohort, e.g. by providing help to endogenous immune cells, remains to be determined.

The efficient recognition of LCL by virion antigen-specific T cells [19] and the correlation of target cell recognition and prolongation of mouse survival implied that increasing virion antigen-specific CD4+ T cells in T-cell preparations might increase their tumor-protective potential. This notion was supported by immunohistochemical analyses of tumor sections which revealed that approximately 1–3% of the tumor cells expressed BZLF1 (data not shown). A similar percentage of BZLF1-positive cells was detected in the corresponding LCL cultures, suggesting that spontaneous induction of the lytic cycle and expression of lytic cycle antigens was not altered *in vivo*.

However, LCL-stimulated T-cell lines were not more tumor-protective at later than at earlier passage. This was either because (i) functionality of the T cells *in vivo* declined with longer *in vitro* culture [31], or (ii) tumor-protective T-cell specificities were lost and only partially compensated for by the increase in virion antigen-specific T cells, and/or (iii) tumor-promoting T cells were enriched. To further analyze antigen-specificity and antitumoral efficacy of early passage T-cell preparations, we compared the tumor-protective potential of T-cell lines stimulated with LCL that had been cultured under different conditions, including those used in clinical protocols [43]. These experiments revealed that potentially autoantigen-specific, but not FCS-reactive or virus-specific T cells, were the principal effectors against PTLD in early passage LCL-stimulated CD4+ T-cell lines. These T cells prolonged mouse survival as effectively as a virion antigen-specific T-cell clone, implicating these two specificities as critical CD4+ effectors against PTLD in this preclinical model. However, one has to keep in mind that only a limited number of T-cell clones directed against a subset of all viral antigens was included in this analysis. Thus, additional T-cell specificities with protective efficacy may exist.

That autoantigen-specific CD4+ T cells are a major component of early passage LCL-stimulated T-cell preparations, has already been demonstrated in earlier studies [20]. Furthermore, when the expansion of lytic cycle antigen-specific T cells was prevented by using LCL Z(-) cells as stimulators, the resulting CD4+ T-cell lines targeted cellular but not viral antigens [21].

Although the antigens recognized by these T cells have yet to be defined molecularly, their expression appears to be restricted to transformed B-cell lines and was not detected in primary hematopoietic cells (Figure S4 in Text S1). In accordance with this, Long et al recently isolated CD4+ T-cell clones from LCL-stimulated lines that recognize cellular antigens expressed in EBV-transformed, but not in mitogen-activated B lymphoblasts [21]. These findings may provide an explanation for the proven clinical safety of LCL-stimulated T-cell preparations [5], [6], [44]. In addition, these findings raise the intriguing possibility that EBV-positive lymphomas that fail to express immunodominant antigens of EBV, e.g. Hodgkin's and Burkitt's lymphoma, and even EBV-negative B cell malignancies, might respond to LCL-stimulated T-cell preparations.

Circumstantial evidence for a protective role of autoreactive CD4+ T cells has already been obtained in preclinical lymphoma models and lymphoma patients: CD4+ T cells that recognize non-viral antigens can prevent B cell lymphomas in mice transgenic for the EBV latent membrane protein LMP1 [45], and five of six patients with Hodgkin's-like and Burkitt's-like post-transplant lymphoproliferative disease responded to treatment with allogeneic T-cell preparations, although in some cases the tumor cells did not express the viral antigens recognized by the infused T cells [11]. Moreover, complete remissions were achieved in several patients with LMP2A-positive Hodgkin's lymphoma by the adoptive transfer of autologous LCL-stimulated T-cell lines. Since the infused T cells contained only low amounts of LMP2A-specific CD8+ T cells and their frequencies failed to correlate with clinical responses [5], [46], additional and still unknown specificities might have contributed to tumor rejection.

Taken together, these results implicate virion and non-viral antigens as important targets of the CD4+ T-cell response against PTLD, and LCL-stimulated T-cell lines, although increasingly replaced by antigen-specific preparations [8], [9], [47], as more potent than previously recognized. Defining the antigens recognized by these non-viral antigen-specific CD4+ T cells and incorporating such specificities in clinically used T-cell preparations may not only increase their antitumoral activity against PTLD, but possibly also against EBV-negative B cell malignancies.

## Materials and Methods

### Ethics statement

All animal experiments were performed in strict accordance with German animal protection law (TierSchG) and approved by the responsible state office Regierung von Oberbayern (ROB) under protocol number 55.2-1-54-2531-131-07. The mice were housed and handled in accordance with good animal practice and all efforts were made to minimize suffering as defined by Federation of European Laboratory Animal Science Associations (FELASA) and the national animal welfare body Gesellschaft für Versuchstierkunde - Society for Laboratory Animal Science (GV-SOLAS).

### Generation and cultivation of LCL

LCL were established by infection of primary B cells with wild-type (wt)-EBV produced by the B95.8 marmoset cell line. MiniLCL and LCL Z(-) were generated by infection of B cells with the genetically engineered virus mutants miniEBV [48] and ΔBZLF1-EBV [49] that are incapable of lytic replication, as previously described [19]. B cells were obtained from peripheral blood mononuclear cells (PBMC) of healthy adult volunteers after informed consent. LCL were cultured as described [20]. In some experiments, FCS was replaced by pooled human serum (HS) to avoid the expansion of FCS-reactive T cells. Where indicated, LCL treated with 200 µM acyclovir (ACV) (Hexal) for at least two weeks were used as T-cell targets.

### Generation and cultivation of T cells

PBMC were repeatedly stimulated with autologous, irradiated (80 Gy) LCL, miniLCL, or LCL Z(-) as antigen presenting cells (APC) as described [20]. Where indicated, T-cell lines were separated into CD4+ and CD8+ fractions by using αCD4+ and αCD8+ MicroBeads, LS-MACS columns and MidiMACS separator as recommended by the manufacturer (Miltenyi Biotec). Purity of the cells was confirmed by FACS analysis using CD3, CD4, and CD8-specific antibodies (Becton Dickinson). Generation and cultivation of CD4+ T-cell clones has been described previously [19], [30]. Clonality of the T cells was assessed by PCR using Vβ chain-specific primers as described, and T-cell epitopes as well as the restricting HLA-molecules were identified using published methods [13], [50]. To exclude that prolonged culture caused loss of specificity of the T cells and, consequently, that their anti-tumor effect *in vivo* would not reflect their initial anti-tumor activity *in vitro*, antigen-specificity of all clones was verified prior to injection (data not shown and Fig S4 in Text S1).

The T-cell lines were generated by stimulation with autologous LCL or miniLCL. The T-cell lines were 100% CD3+ with varying proportions of CD4+ and CD8+ components. No NK or B cells were detected by FACS. Target cell recognition and lytic activity of all T cells was tested prior to injection (data not shown). Cytokine secretion by the T cells was measured by ELISA (R&D Systems). Plotted data represent the mean plus standard deviation (SD) of triplicates. Dendritic cells and PHA blasts were generated as described [51]. Cytolytic activity was measured after 3 h of co-culture of T cells with labeled target cells by quantitating calcein AM (Invitrogen) released into the culture supernatant. Virus concentrate was prepared by ultracentrifugation of B95.8 cell culture supernatant. Functionality was tested using BLLF1-specific T cells (Figure S3 in Text S1) and viral copy numbers determined by qPCR as described [13].

### *In vivo* studies

To assess the antitumoral potential of T cells *in vivo*, 1×10^7^ LCL (LCL-SCID mouse model) or 5×10^7^ PBMC (PBMC-SCID mouse model) from EBV-positive donors were injected intraperitoneally (i.p.) into 6 to 14-weeks-old C.B.17-SCID mice (Taconic). 1×10^7^ T cells in PBS, or PBS only, were i.p. injected separately on the same day before down-regulation of HLA class-II on injected LCL occurs [40], [52]. All cells injected in mice were tested negative for mycoplasma using a commercial detection kit (Lonza). For T-cell tracking experiments, LCL were injected on day 0 and T cells on day 25. Experimental groups consisting of 4–6 mice were evaluated for tumor growth and survival. Mice were sacrificed when they had ruffled hair, showed food refusal, bulky abdomen or palpable tumors. To verify the presence of human B cells in these mice, human IgG (huIgG)-ELISA was performed. 96-well plates were coated with α-human IgG mAb (2.5 µg/ml; Abcam) in PBS overnight and then incubated with mouse serum at different dilutions in RPMI-1640 for 1 h. Subsequently, the biotin-labeled detection antibody α-huIgG (Dianova) was added for 1 h followed by horseradish-peroxidase (HRP)-coupled streptavidin for 20 min. HuIgG was visualized by adding TMB-substrate.

Where indicated, T cells were labeled with CFSE according to the guidelines of the manufacturer (Invitrogen). For the FACS-analysis of tumor infiltration by CFSE-labeled T cells, single cell suspensions of tumors were prepared by mechanical disruption and lysis of erythrocytes.

### FACS

For FACS analysis, fluorochrome-conjugated monoclonal antibodies against human CD3, CD4, CD8, CD25, CD28, CD57, CD62L, CXCR3, CCR4, CCR6, CCR7, CTLA-4, MHC II, PD-1 (Becton Dickinson), CD20, CD27 and MHC I (ImmunoTools) were used. TIM-3 antibody (kindly provided by Dr. Kuchroo, Boston) was visualized using a fluorochrome-conjugated secondary antibody (Jackson ImmunoResearch laboratories). Granzyme A and B stainings were performed on α-CD3-activated T cells. Dead cells were excluded with 7-AAD (Becton Dickinson), cells fixed with paraformaldehyde, permeabilized with saponine and stained for granzyme A and B. FoxP3 staining was performed following the manufactureŕs protocol using the Fix/Perm FoxP3 buffer set (BioLegend). CD4+ cells were stained prior to fixation, CD25+ cells were stained simultaneously with FoxP3. CD107a antibody (BioLegend) was added during T-cell stimulation and surface expression analyzed after 4 h. Flow cytometric analysis was performed in a FACSCalibur flow cytometer and data analyzed with the CellQuest software (Becton Dickinson).

### Immunohistochemistry

Immunohistochemical analyses were performed on cryo-embedded or formalin-fixed, paraffin-embedded (FFPE) tumor samples. FFPE-sections of all tumors were stained with hematoxylin and eosin (H&E), or with antibodies against human CD20, EBNA1, EBNA2, BZLF1, BLLF1, and FITC (from Argene, Dako, or kindly provided by Dr. E. Kremmer, Helmholtz Zentrum München). For H&E staining, FFPE sections were stained with mayeŕs hematoxylin solution and eosin Y (both Roth). Single stain immunohistochemistry was performed on FFPE sections using the Vectastain ABC Detection System for horseradish peroxidase according to the manufactureŕs protocol (Vector Laboratories). Cryo-embedded sections were used for double-stainings with antibodies against FITC, to detect CFSE-labeled T cells, and BLLF1, to detect lytically infected tumor cells. In addition to the horseradish peroxidase detection system, the Vectastain ABC Detection System for alkaline phosphatase in combination with the alkaline phosphate substrate kit III (both from Vectastain) was used.

### Statistical analysis

Mouse survival was analyzed using Kaplan-Meier curves. Significances of the *in vivo*-experiments were calculated by using the log-rank or the Kruskal-Wallis test. p-values of 0.05 or less were considered significant. The statistical analyses were carried out with the GraphPad Prism 5 program.

## Supporting Information

Text S1 Supporting information. This file contains Figures S1-S5 and Table S1.
(DOC)
