# Supplementary material for: Virus and Autoantigen-Specific CD4+ T Cells Are Key Effectors in a SCID Mouse Model of EBV-Associated Post-Transplant Lymphoproliferative Disorders
Source: PLoS Pathog. 2014 May 22;10(5):e1004068. doi: 10.1371/journal.ppat.1004068 (PMC4031221; doi:10.1371/journal.ppat.1004068)

**Supporting information**

**Table S1**

Listing of the injected T cell clones and lines, their antigen specificities, and their effects on tumor growth in mice (“+/-“, mouse survival affected +/- 1 day; “+“, mouse survival prolonged for 2 - ≤10 days; “++”, mouse survival prolonged for >10 days; “-“, mouse survival shortened for 2 - ≤10 days). n.s.; not significant.

| **Figure** | **T cell line** | **T cell clone** | **Specificity** | **Effect on tumor growth** | **Significan**c**e**  **[p-value]** |
| --- | --- | --- | --- | --- | --- |
| 3A |  | EBNA1-1C3 | Latent antigen | +/- | n.s. (0.6374) |
| 3D |  | EBNA1-3E10 | Latent antigen | - | <0.0001 |
| 3A |  | EBNA3-3B | Latent antigen | - | n.s. (0.5416) |
| 3A |  | EBNA3C-3H10 | Latent antigen | + | n.s. (0.0879) |
| 3A |  | BLLF1-1D6 | Late lytic antigen | + | 0.0034 |
| 3D |  | BNRF1-1H7 | Late lytic antigen | - | <0.0001 |
| 4B | T cell line p4 |  | unknown | ++ | <0.0001 |
| 4B | T cell line p10 |  | unknown | ++ | <0.0001 |
| 5B | T cell line HS |  | unknown | ++ | <0.0001 |
| 5B | T cell line FCS |  | unknown | ++ | <0.0001 |
| 5B | T cell line FCS-ACV |  | unknown | ++ | <0.0001 |
| 5C | T cell line HS |  | unknown | ++ | <0.0001 |
| 6B | JM-W3 |  | non-viral | + | 0.0117 |
| 6B | GB-W3 |  | non-viral | + | <0.0001 |

**Supporting information legends**

**Figure S1**

**Efficacy *in vivo* does not correlate with lytic activity of the T cells.**

(A) LCL Z(-) were pulsed with increasing concentrations of cognate peptide for 1 hour. Following extensive washing to remove unbound peptide, the cells were co-cultured with BNRF1-1H7 and BLLF1-1D9 T cells and cytokine secretion (IFNγ, GM-CSF, IL2 and IL4) was measured 20 hours later. (B) Target cells pulsed with 1 µM of cognate or control peptide were incubated with the T cells at different target to effector ratios. Cytolytic activity of T-cell clones was measured after 3 hours (*** p<0.0001).

**Figure S2**

**Characterization of T-cell lines at passage 4 *versus* 10.**

(A) Clonality of T-cell lines at passage 4 and 10 was analysed by PCR using primers specific for 25 different Vß chains (numbers above agarose gels). PCR products were separated on an ethidium bromide-stained agarose gel (M; size marker). Actin served as positive (+) and H_2_O instead of cDNA as negative (-) control for PCR. (B) The cytolytic potential of the T-cell lines was assessed by FACS analysis of the degranulation marker CD107a, and of granzyme A and B (filled graph: isotype control). (C) Lytic activity of the T-cell lines against autologous LCL was assessed after 3 hours of co-culture. PHA blasts of the same donor served as controls. (D) FACS analysis of the T-cell exhaustion markers CD57, CTLA-4, TIM-3, and PD-1 (filled graph; isotype control).

Except for a slight reduction in the Vß chain repertoire of the T-cell line at passage 10, only marginal differences in the expression of degranulation markers, exhaustion markers, and lytic activity were observed between these two lines.

**Figure S3**

**T-cell lines generated by stimulation with LCL FCS-ACV fail to recognize lytic cycle antigens.**

T cells stimulated with autologous LCL grown in media containing FCS and acyclovir (ACV) were co-cultured with autologous LCL FCS-ACV, LCL, LCL Z(-), and LCL Z(-) that had been pulsed with EBV viral particles (LCL Z(-) + virus). IFNγ secretion by the T cells was determined 20 hours later. Efficient recognition of LCL FCS-ACV and LCL Z(-) indicated that these T cells were not specific for lytic cycle antigens of EBV. Consistent with this, recognition of LCL Z(-) was not enhanced when the cells had been virus-pulsed. BLLF1-1D6 T cells recognizing a peptide derived from the virion protein gp350 were used to verify that LCL FCS-ACV and LCL Z(-) lacked expression of late lytic cycle proteins, and that these target cells were able to stimulate virion-specific T cells when loaded with virus or specific peptide.

**Figure S4**

**The T-cell lines JM-W3 and GB-W3 recognize non-viral antigens.**

(A) PBMC of donor JM were pulsed with recombinant latent proteins of EBV (500 ng/ml) for 24 hours. After extensive washing, the cells were probed with the indicated EBNA-specific CD4+ T-cell clones and IFNγ secretion was measured 20 hours later by ELISA. In the case of LMP1 and LMP2A, against which no specific CD4+ T cells were available, the epitope recognized by EBNA3C-specific T cells was fused to the C-termini of LMP1 and -2A. All T-cell clones specifically recognized target cells pulsed with their cognate antigen. Recognition of recombinant EBNA3A by specific T cells was verified in a separate experiment (data not shown). Thus, the failure of GB-W3 CD4+ T cells to recognize target cells pulsed with latent antigens is not due to an inefficient processing or presentation of these proteins. (B) GB-W3 CD4+ T cells were co-cultured with autologous (GB), HLA-matched (JM), or mismatched (UB) dendritic cells and LCL. In addition, the HLA-matched, EBV-negative Hodgkin lymphoma cell lines L428 and HDLM2 were used. T cells alone (w/o) served as negative control. (C) JM-W3 CD4+ T cells were co-cultured with autologous PBMC, dendritic cells (DC), or LCL established by infection with different EBV strains (B95.8, EBV type I; Ag876, EBV type II; miniEBV; BZLF1 knock-out (k.o.) EBV) as well as LCL that spontaneously grew out from peripheral blood of the donor *in vitro* (LCL spontan). Furthermore, HLA-matched (MSC) and mismatched (DA) LCL, as well as the HLA-matched EBV-negative BL30 cell line and the EBV-positive convertants of this cell line (BL30-B95.8 and BL30-P3HR1) were used. The exclusive recognition of transformed cells by these T cells, irrespective of EBV-status, indicates that these T cells recognize non-viral antigens. (D) To investigate whether BL30 cells are capable of processing and presenting antigen on MHC class II, the indicated target cells were incubated with increasing amounts of recombinant influenza M1 protein for 24 hours and subsequently probed with M1-specific CD4+ T cells . LCL JM served as positive control. All three protein-pulsed BL30 cell lines were recognized by the M1-specific T cells with similar efficiency. Thus, the failure of JM-W3 cells to recognize BL30-B95.8 and BL30-P3HR1 cells is not because of an antigen processing or presentation deficiency. Rather, these results suggest that these T cells recognize autoantigens that are expressed to variable degrees by transformed, but not untransfomed cells.

**Figure S5**

**Injected T-cell clones and lines lack FoxP3 expression.**

FoxP3 expression in T-cell lines and clones was assessed by intracellular FACS. Depicted cells were gated on CD4+CD25high. As exemplified by two T-cell clones and one T-cell line, no FoxP3 expression was detected. A regulatory T-cell line served as positive control.

**Figure S1**


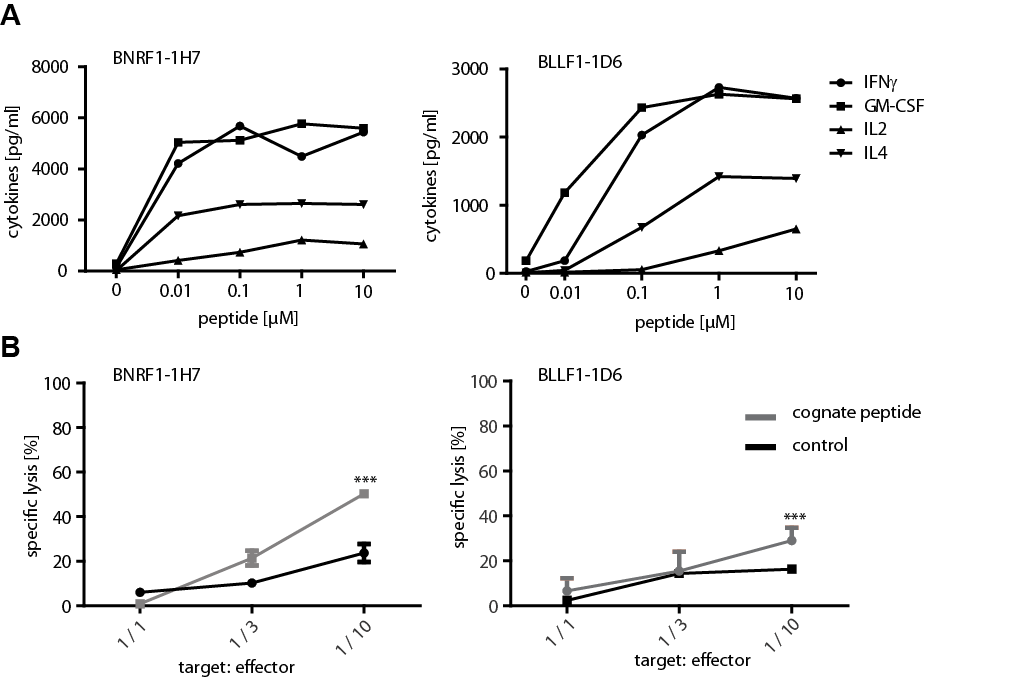


**Figure S2**


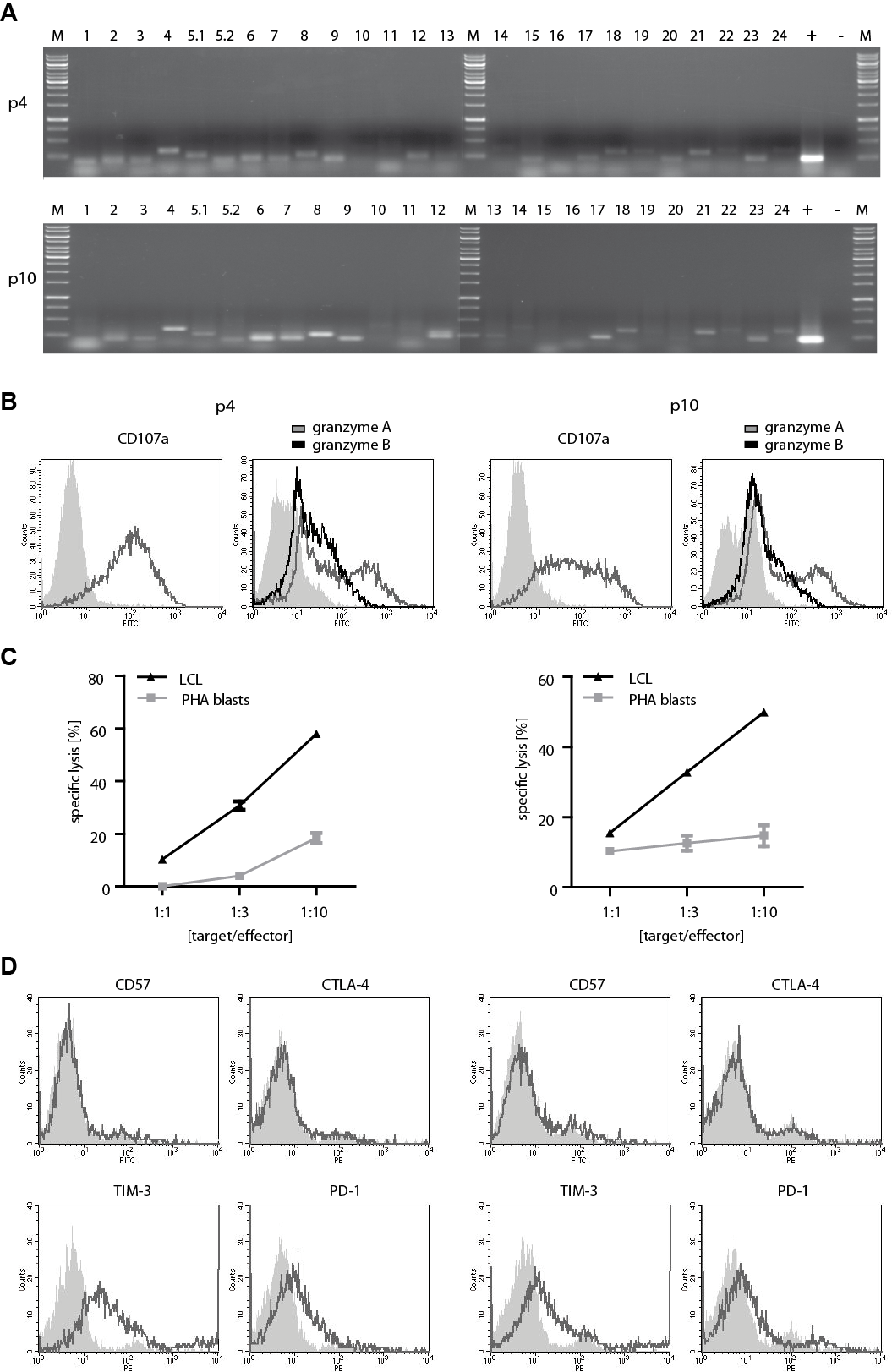


**Figure S3**


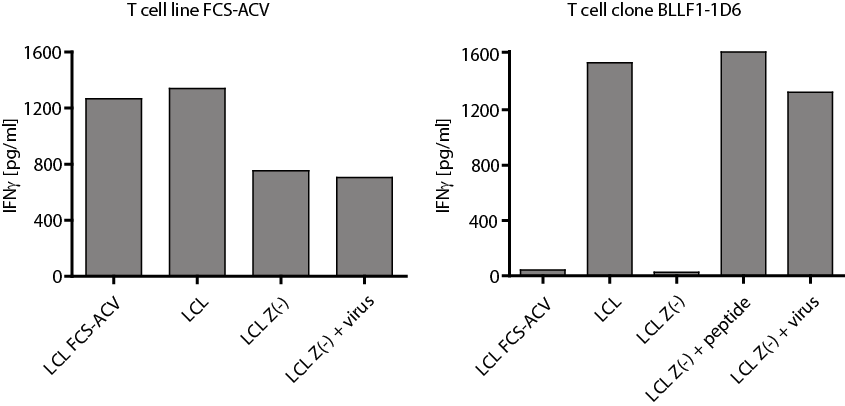


**Figure S4**


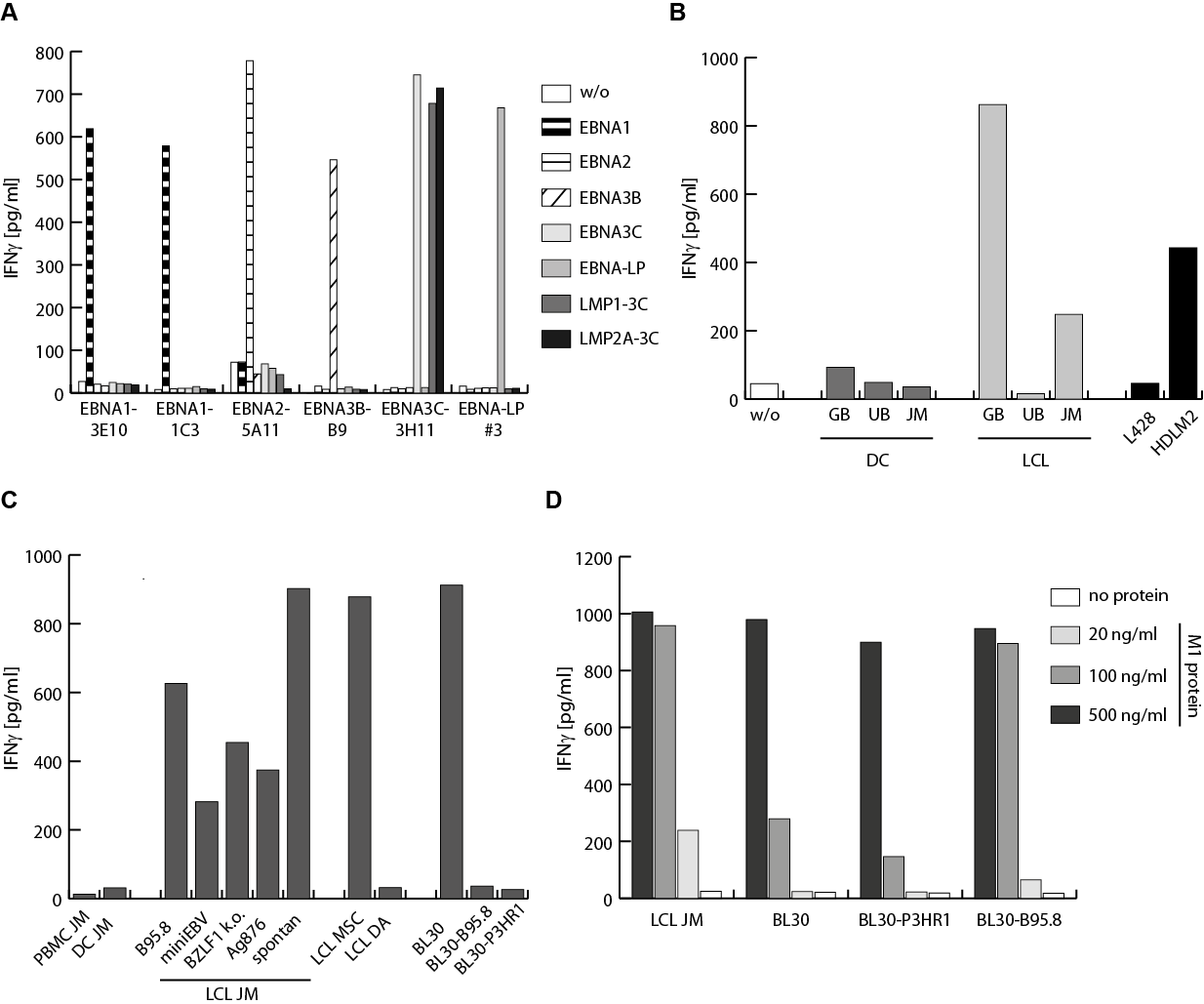


**Figure S5**


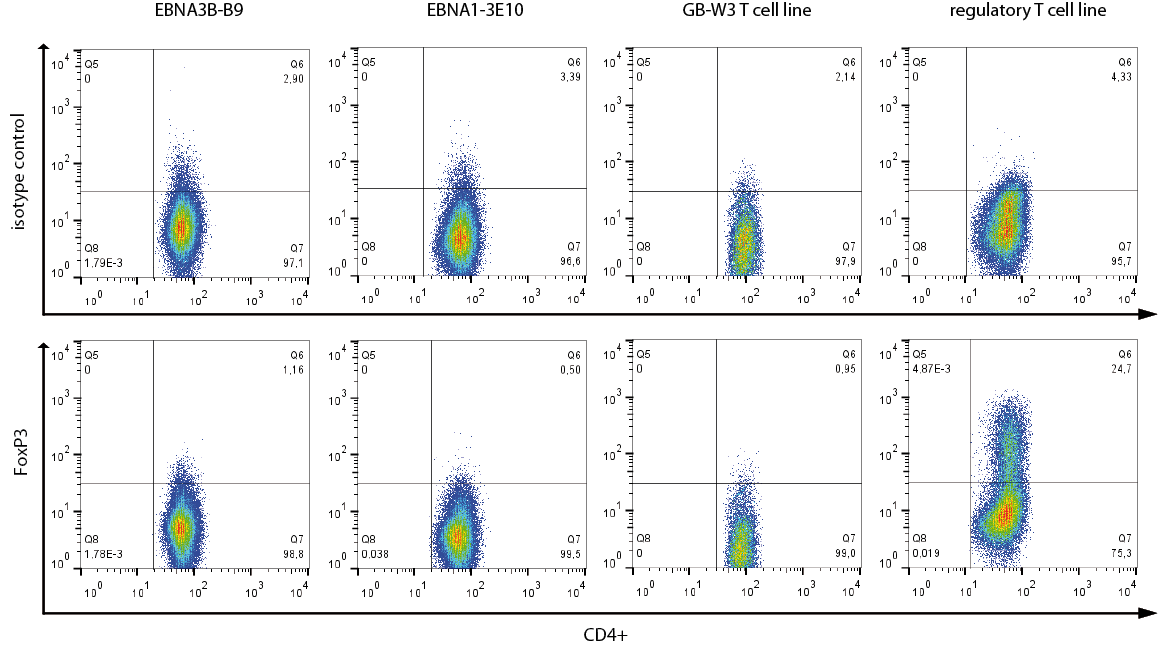

Supplement: Text S1 — Supporting information. This file contains Figures S1-S5 and Table S1. (DOC) [file ppat.1004068.s001.doc]
